# Maintenance of Complex Trait Variation: Classic Theory and Modern Data

**DOI:** 10.3389/fgene.2021.763363

**Published:** 2021-11-12

**Authors:** Evan M. Koch, Shamil R. Sunyaev

**Affiliations:** ^1^ Department of Biomedical Informatics, Harvard Medical School, Boston, MA, United States; ^2^ Division of Genetics, Brigham and Women’s Hospital, Harvard Medical School, Boston, MA, United States

**Keywords:** population genetics, genome-wide association study, statistical genetics, evolution, quantitative genetics

## Abstract

Numerous studies have found evidence that GWAS loci experience negative selection, which increases in intensity with the effect size of identified variants. However, there is also accumulating evidence that this selection is not entirely mediated by the focal trait and contains a substantial pleiotropic component. Understanding how selective constraint shapes phenotypic variation requires advancing models capable of balancing these and other components of selection, as well as empirical analyses capable of inferring this balance and how it is generated by the underlying biology. We first review the classic theory connecting phenotypic selection to selection at individual loci as well as approaches and findings from recent analyses of negative selection in GWAS data. We then discuss geometric theories of pleiotropic selection with the potential to guide future modeling efforts. Recent findings revealing the nature of pleiotropic genetic variation provide clues to which genetic relationships are important and should be incorporated into analyses of selection, while findings that effect sizes vary between populations indicate that GWAS measurements could be misleading if effect sizes have also changed throughout human history.

## 1 Introduction

Attempts to understand genetic architecture preceded the discovery of DNA as the model of heredity (Fisher, 1918), and much theoretical work on selection, the maintenance of variation, and the adaptation of complex traits began before the ability to record genotypes on a scale sufficient to meaningfully contribute to these questions (Walsh and Lynch, 2018). The modern genetic era has provided an opportunity to test classic theories and to expand models—both long-standing and relatively recent—based on new understandings of genetic architecture and mechanisms. Genome-wide association studies (GWAS) and other data-driven tools have raised additional questions, including how so much heritability for many traits is contributed by relatively common alleles when natural selection is often expected to remove deleterious variation from the population. The flood of methods and data has sharpened and revised our understanding of many components that fashion the structure of the genome—polygenicity, selection, the distribution of mutational effects, pleiotropy—but has left us wanting for models capable of reconciling these elements (Sella and Barton, 2019).

The analysis of GWAS data revealed an extraordinary degree of polygenicity, and showed that most heritability is explained by relatively common, mostly noncoding alleles of small effect. At first glance, this observation is surprising. Natural selection is expected to maintain the population near an optimum value for quantitative traits and to reduce the prevalence of potentially maladaptive phenotypes such as diseases. Such optimums and maladaptive phenotypes are defined within a given environmental context (Harpak and Przeworski, 2021). Selection generally acts by reducing the frequency of phenotypically relevant alleles, though it may drive allele frequency increases when shifts in the optimum phenotype occur. This basic logic led to the question whether the effect of natural selection is evident from GWAS data at all. Recent studies have reached a strong consensus that phenotypic effect sizes are negatively correlated with allele frequency (Gazal et al., 2018; Zeng et al., 2018; Schoech et al., 2019; Speed et al., 2020; Zeng et al., 2021). These findings are inconsistent with purely neutral models, but various models of natural selection influencing trait variation remain plausible (Walsh and Lynch, 2018). Uncertainty largely surrounds whether the focal trait is causally important for fitness compared to pleiotropically related ones, and whether selection is primarily stabilizing or has important directional components. In spite of many unresolved details, the emerging picture is that a vast supply of mutations with weak effects, coupled with generally inefficient selection against such alleles, is the basis of phenotypic variation.

Empirical results from GWAS on the distribution of effect sizes and allele frequencies still pose the challenge of which classic and emerging models from theoretical population genetics are able to best explain the emerging observations. Existing theories range from models of selection acting directly on the focal trait to models where selection on genetic variation is driven by simultaneous effects on other traits (pleiotropy), to even fully “apparent” selection, which assumes the focal trait is not subject to any selective constraint. In this review, we discuss a relevant subset of these models and how their predictions look in light of recent studies of selection in GWAS. We identify pleiotropy and variable effect sizes of genetic variants across time and space as important factors that have yet to be satisfactorily included into statistical methods and theoretical models.

## 2 Theoretical Models of Maintenance of Complex Traits and Predictions They Generate

Evolutionary quantitative genetics has subsisted for most of its existence on a limited set of possible measurements. Estimates of the genetic and mutational variance, as well as selection gradients, are informative, especially with respect to contemporary patterns of selection. However, most progress in explaining maintenance of genetic variation in phenotypic traits was theoretical. Now that GWAS have generated an abundance of matched phenotypic and genetic measurements we live in a much more data-rich world. If we turn our attention to a single, focal trait, what sort of data would we ideally wish for? We would probably include the impact of genetic variants (estimated as their effect size) on the trait in a range of environments, the fitness effects of these alleles, as well as their frequencies and linkage patterns (Johnson and Barton, 2005). These would yield a satisfying and useful description of the genetic architecture and the process of its development, but there are fundamental details not immediately obvious from this description. We would like to know whether fitness effects arise primarily through selection on the focal trait, and if so what form it takes. If there are substantial fitness effects unrelated to the focal trait, what other traits are involved and how does selection act on them? Is the population in equilibrium? Has the genetic architecture changed in the past and will it do so in the future? Questions like these can be addressed by modeling how selection acts on traits, the mutational distributions underlying them, and how these generate the genetic architectures we observe.

Textbook introductions to population genetics begin by assigning fitnesses to genotypes and examine the consequences for allele frequencies and overall patterns of genetic variation. Connecting trait values, such as those measured in GWAS, to selection on individual causative alleles requires the additional step of specifying how selection on phenotypes leads to fitness differences among genotypes. While slightly less familiar than other selection results, this task was also taken up by many of the authors of classical population genetics and has grown into a large branch of evolutionary theory.

**FIGURE 1:**
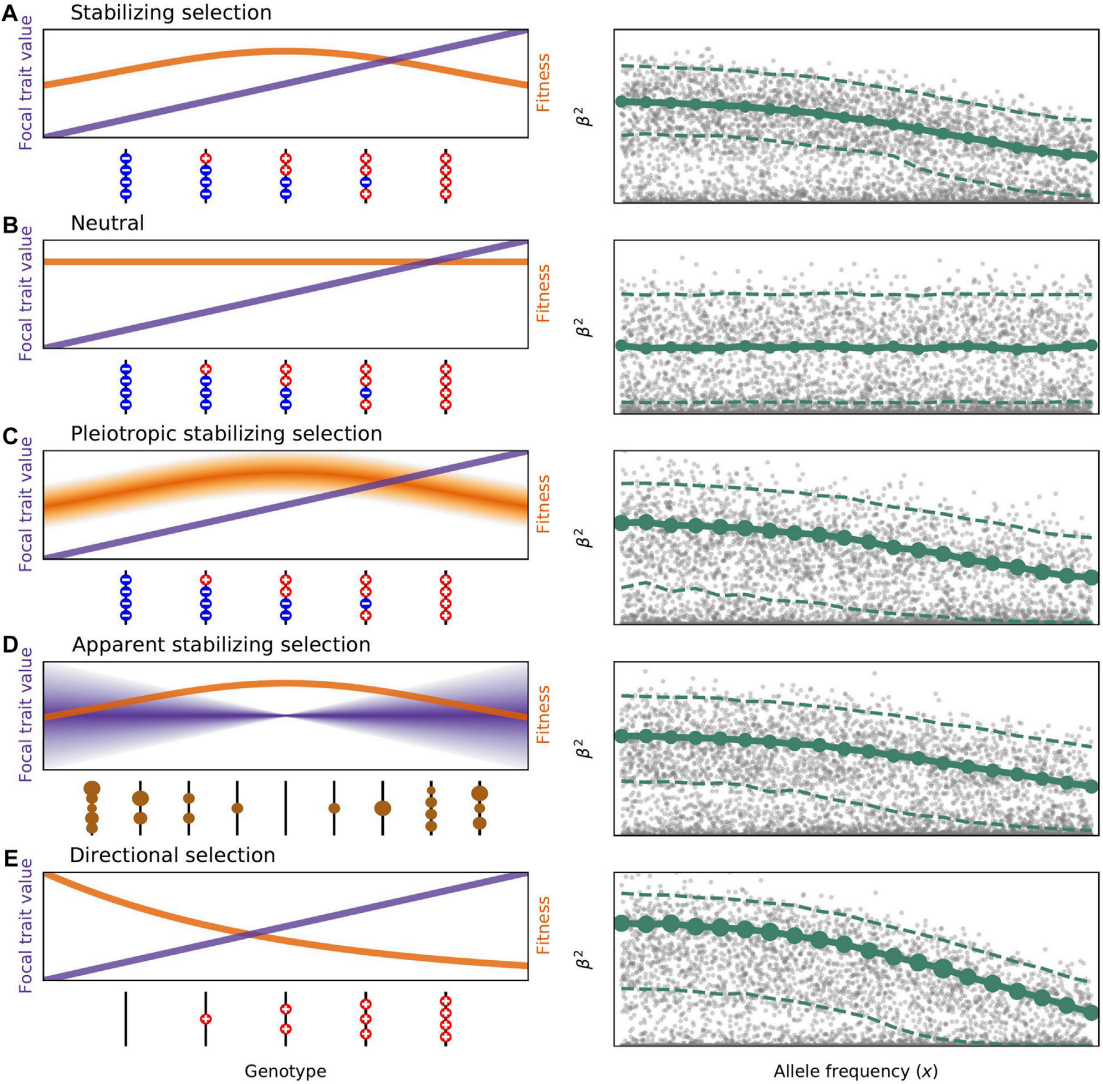
Models of selection on the genetic variation influencing complex traits. Panels on the left show how different genotypes affect trait values (in purple) and fitness (in orange). Panels on the right illustrate how squared trait values change with frequency in each model of selection. Simulated values are shown in grey, the mean *E*[*β* ^2^|*x*] is represented by the solid green line, and the standard deviation of *β* ^2^|*x* is represented by the size of the green circles. The median and 97.5*%* quantile are shown as dashed lines to give a better sense of the full leptokurtic distribution of effect sizes. The DFE used in all plots (shape = 0.25, scale = 40) was chosen to be within the range fit by Schoech et al. (2019). Effect sizes were simulated uniformly on log frequency, and both axes are on a log scale. **(A)** Classic stabilizing selection as described by Eq. 1. Genotypes containing more trait-increasing alleles than decreasing, and vice versa, have lower fitness as a result of selection on the focal trait. Large effect alleles are prevented from reaching high frequencies due to the variance-reducing property of stabilizing selection. **(B)** In the neutral model no genotype is more fit than any other and the distribution of effect sizes at any frequency reflects only the distribution of mutational effects. **(C)** In pleiotropic stabilizing selection as studied by Simons et al. (2018), there is variation in fitness for each genotypic values depending on the effects mutations have on pleiotropic traits. This leads to the same average *E*[*β* ^2^|*x*] but a greater variance and therefore different genetic architecture. **(D)** Models of apparent stabilizing selection first specify the deleterious fitness effects of mutations, represented here by the size of the brown circles. Genotypes with more and stronger deleterious mutations have a greater variance in phenotypic outcomes. This too leads to a negative relationship between *β* ^2^ and *x*. Here we use the Eyre-Walker (2010) model with *τ* = 0.4 as fit by Schoech et al. (2019), and *σ* ^2^ = 1. Altering these would change the mean and variance of the (*β* ^2^, *x*) relationship. **(E)** Directional selection is shown here for a scenario where trait-decreasing mutations are unlikely or impossible. Selection therefore acts to reduce the frequency of trait-increasing alleles. All new mutations are disfavored with *s* ∝ *β*. *E*[*β* ^2^|*x*] again decreases with *x*.

The simplest and most obvious model predicts the selection on individual causative loci arising from stabilizing selection on a single polygenic trait with purely additive genetic variance (Wright, 1935; Robertson, 1956; Bulmer, 1972) (Figure 1A). In this model an individual’s trait value (*z*) is determined by the sum of effects from *L* independent loci: $z=\sum_{l=1}^{L}\left(\beta_{l}g_{m}+\beta_{l}g_{p}\right)+e$ , where *β*_*l*_ is the effect size of the allele at locus *l*, *g*_*l*_,_*m*_ and *gl*,_*p*_ are the maternal and paternal genotypes at each locus, and *e* is a normally distributed environmental effect centered at zero. If an individual’s fitness is a Gaussian function centered at the population mean *M* and with width *V*_*S*_ (*w*(*z*) = exp (−(*M* − *z*)^2^/2*V*_*S*_)), then selection will change the average frequency of a causative allele at locus *l* with effect size *β*_*l*_ = *β* as follows:

$$\text{E}\left[\Delta x\right]\approx -\frac{\beta^{2}}{2V_{S}}x\left(1-x\right)\left(\frac{1}{2}-x\right).$$ (1)

Stabilizing selection tends to remove genetic variation in this trait from the population. A balance between mutation, selection, and drift generates the trait’s genetic variance in the population (Bulmer, 1972; Keightley and Hill, 1988). Such direct stabilizing selection leads to a negative correlation between minor allele frequencies and the effect size magnitude.

We can write the selection coefficient for this model as $s_{ud}=-\frac{\beta^{2}}{2V_{S}}$ to acknowledge that stabilizing selection takes the underdominant form shown in Eq. 1 ( $\text{E}\left[\Delta x\right]=sx\left(1-x\right)\left(\frac{1}{2}-x\right)$ ) rather than the more familiar additive one (E[Δ*x*] = *sx* (1 − *x*)). The $\left(\frac{1}{2}-x\right)$ term appearing in the underdominant formula means that selection against the derived allele actually decreases as it approaches 50*%* frequency and actually switches signs after that point. The minor allele is therefore always disfavored. However, when selection is strong or the allele frequency is low, the differences are minor as *x* is small compared to 1/2. We generally omit the subscript in *s*_*ud*_ for ease of reading, but it is important to note that the interpretation of selection coefficients differs depending on whether stabilizing selection is explicitly modeled or not.

The variance-reducing property of stabilizing selection motivated the development of other models with variance-promoting features like overdominant side-effects of causative alleles (Robertson, 1956; Gillespie, 1984) and strong mutational pressure (Lande, 1976b). As always in evolution, we must also at least consider the possibility that a trait of interest has a negligible impact on organismal fitness. The population mean value of a trait controlled by strictly neutral mutation will drift in Brownian motion and have a genetic variance that depends on the mutation rate, the second moment of the distribution of mutation effect sizes, and the average pairwise coalescent time between randomly sampled loci (Lande, 1976a; Lynch and Hill, 1986; Koch, 2019).  Var[*z*] = E[*T*_2_]*θμ*_2_, where *T*_2_ is the average number of generations it takes for a pair of sites to coalesce, *θ* is the mutation rate per generation, and *μ*_2_ is the second moment of the distribution of mutational effects. Crucially, there would be no relationship between the effect size and frequency of alleles (Figure 1B).

Of course, both intuitively and empirically, traits in natural and contemporary human populations at least appear to be under some selection (Kingsolver et al., 2001; Corbett et al., 2018; Sanjak et al., 2018), and involve some level of pleiotropy (Stearns, 2010). Models of apparent selection begin with the assumption that the focal trait is not itself under any selection but add pleiotropic fitness effects of the causative alleles. Individuals in the tails of a phenotypic distribution will carry more mutations overall, and if trait-affecting mutations have deleterious pleiotropic effects those individuals will also have lover fitness on average (Barton, 1990; Kondrashov and Turelli, 1992). Fitness that decreases away from the mean is reminiscent of stabilizing selection, but the strict deleterious model of apparent selection does not induce the negative correlation between allele frequencies and effect size magnitudes expected when the focal trait itself is actively selected. The negative correlation between *β* ^2^ and *x* may yet be rescued if the deleterious pleiotropic effects of variants affecting the focal trait arise from genetic covariance (Lande and Arnold, 1983) or correlated effect size magnitudes (Keightley and Hill, 1990). In this scenario, alleles with larger effects (or absolute magnitudes) on the focal, neutral trait are more likely to have larger effects on a second, selected trait. Allele frequencies are suppressed through selection on the second. In the extreme where the focal and selected trait are so closely biologically related that the effect sizes of mutations are deterministically linked, it is indistinguishable which trait causally impacts fitness, although a strong genetic covariance would be measurable. One can also imagine a model where each mutation has such a relationship with a unique pleiotropic trait, for instance, molecular effects in different pathways. Assuming that large-effect alleles for the focal trait induce stronger molecular effects, there may be strong selection without measurable genetic covariance between the focal trait and any individual pleiotropic trait.

The differences between models come down to how the statistical relationship between selection coefficients and effect sizes is specified: how *s* scales on average with *β* and what the random variation around this looks like. In multivariate stabilizing selection, *s* scales with *β* ^2^ as in direct stabilizing selection, but apparent selection models don’t have this restriction. Apparent selection models were extended, as described above, to include increasing selection with greater *β* in addition to the negative pleiotropic consequences (Keightley and Hill, 1990; Zhang and Hill, 2002; Eyre-Walker, 2010) (Figure 1D). Models of multivariate stabilizing selection paint a similar picture, but the focal and pleiotropic traits are explicitly under stabilizing selection (Zhang and Hill, 2003; Simons et al., 2018) (Figure 1C). All lead to a negative (*β* ^2^, *x*) relationship, so differences between models come down to the shape and variance of that relationship, along with impacts on the genetic architecture.

Directional selection on complex disease susceptibility is also a viable hypothesis. In this view, the disease phenotype is itself deleterious and alleles that increase susceptibility will be selected against (Charlesworth, 2001; Wright et al., 2003) (Figure 1E). This also implies a mutational bias towards susceptibility-increasing alleles. It is plausible that there is a fitness cost associated with carrying such alleles, even for late-onset diseases (Pavard and Coste, 2021). All of the pleiotropy arguments made for stabilizing selection would apply equally well here.

There is an emerging consensus that models of mutation-selection-drift balance are likely to explain the genetic architecture of many, if not most, complex traits (Sella and Barton, 2019). The models of apparent, stabilizing, and directional selection described above, with varying possible degrees of pleiotropic selection, all remain possibilities within this consensus and are not mutually exclusive. Progress in statistical genetics methodology and increasing GWAS sample sizes are starting to clarify these details.

## 3 Detecting Negative Selection in Genome-Wide Association Studies Data

As sample sizes increased and GWAS became sufficiently powered to detect larger numbers of loci for different traits, attention started shifting from the speculative question of how study design should be informed by selection and its effect on genetic architecture (Pritchard, 2001; Reich and Lander, 2001), to what the genetic architecture, as revealed through these studies, might say about selection. A transitional form was contributed by (Agarwala et al., 2013), who investigated how selection may have shaped the genetic architecture of Type 2 Diabetes, which had recently gone from 2 to 39 genome-wide significant loci. Using primarily the number of associations, conditional on the heritability and prevalence of the disease, they ruled out both neutrality of the focal trait and a model where selection is proportional to effect size: *β* ∝ |*s*|.

Following this, methods were developed that do not explicitly model natural selection on causative variants, but ask whether lower frequency variants contribute disproportionately to heritability. This heritability bias should only occur if rare variants have larger effect sizes on average, the most plausible explanation being negative selection correlated with the magnitude of effect sizes. A simple approach is to divide variants into MAF bins and estimate the heritability contribution of each in a mixed model framework (Yang et al., 2015). applied this approach to height and body mass index (BMI) and (Mancuso et al., 2016) to prostate cancer risk. Both found increased heritability in rare variants compared to common, and Mancuso et al. (2016) performed simulations to demonstrate that, conditional on disease heritability and prevalence, they could also rule out focal trait neutrality and directly proportional selection.

More sophisticated analyses using the same general idea as partitioning heritability by allele frequency have been developed and applied to a wide variety of human traits. Extensions of LD score regression (LDSC), a useful tool for partitioning heritability among large numbers of annotations (Finucane et al., 2015), were developed for features of negative selection. These analyses found that younger genetic variants contribute more heritability than older genetic variants at the same frequency (Gazal et al., 2017), a key feature of negative selection (Maruyama, 1974; Kiezun et al., 2013). They also confirmed earlier findings that rare variants have greater effect sizes than common ones for a larger number of traits (Gazal et al., 2018). Another popular and tractable approach, termed the alpha model, explicitly sets the MAF dependence of heritability contributions through a single parameter *α*: E[*β* ^2^|*x*] ∝ (*x*(1 − *x*))^*α*^ (Zeng et al., 2018; Schoech et al., 2019; Speed et al., 2020; Zeng et al., 2021). An *α* < 0 indicates a heritability bias towards rare variants and some amount of negative selection. Applications of this model have been remarkable in their consistently negative *α* estimates across all analyzed traits. While some differences between traits are inferred, e.g. height has a smaller *α* than BMI, estimates are consistently within the range [− 0.5, −0.2].

**FIGURE 2:**
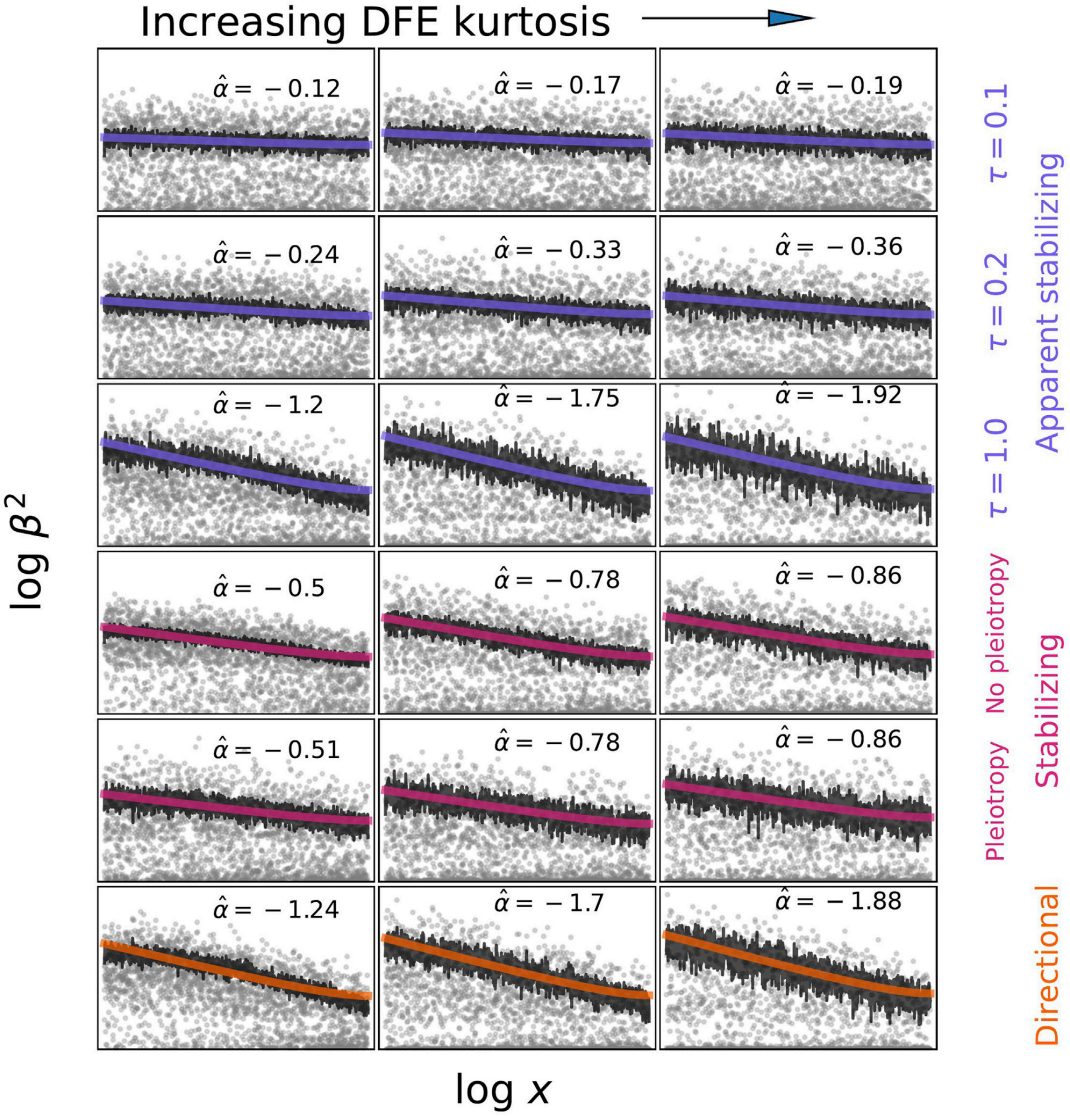
Examples of what alpha models may infer under different models of selection and different distributions of fitness effects (DFE). Effect sizes were simulated by sampling from *p*(*s*|*x*) and then from *p*(*β*|*s*) under the different models described in the text. Derived allele frequencies are uniform between 0.01 and 0.5. Estimates of *α* were obtained by fitting log  *β* ^2^ = *α*  log  *x* (1 − *x*) + *c* to the average *β* ^2^|*x* values calculated from simulations. The DFE was varied by decreasing the shape parameter from 1 to 0.25 to 0.125 while keeping the mean constant. It is important to recognize that $\hat{\alpha }$ values reported here would not necessarily correspond to those obtained by real statistical genetics methods (Zeng et al., 2018; Schoech et al., 2019; Speed et al., 2020; Zeng et al., 2021). Those methods employ particular likelihoods and are applied to real genetic data where frequencies are not uniform and effect sizes are estimated with error. The frequencies of analyzed variants may be particularly important since the slope of the (*β* ^2^, *x*) relationship (local *α*) varies with frequency (Schoech et al., 2019). Estimated *α* values increase with increasing DFE kurtosis, reflecting the proportion of variants that are strongly selected. For high kurtosis, estimates approach the theoretical expectation of *α* = − 2*τ* for the Eyre-Walker (2010) model as derived by Schoech et al. (2019). As expected, in a model of stabilizing selection (Simons et al., 2018), the degree of pleiotropy does not affect the *α* estimate. Directional selection is associated with higher *α* estimates than stabilizing selection.

The negative relationships between effect size magnitude and minor allele frequency inferred for so many traits are informative about the model of selection. In particular, they allow us to rule out neutral models where the focal trait and all underlying variation are unaffected by selection, as well as strict models of apparent selection where the causative variants are deleterious, but the strength of this selection is uncorrelated with effect sizes. However, many other models of selection may still be compatible with these findings (Figure 1, Figure 2). The model of direct stabilizing selection on a single trait first proposed by Wright and others (Equation 1) is plausible for some traits. On the other hand, a genetic correlation between the focal trait and another (or many) under stabilizing selection could produce the observed negative correlations even if the focal traits were completely neutral. There is also a lot of space in between with varying contributions to selection from the focal and pleiotropic traits. While seemingly semantic, the question is really about the extent to which variation in the underlying biology of the focal trait causes variation in fitness. This can apply even when the focal trait is something seemingly benign, an arbitrary bone for instance, whose size is governed chiefly by the biology of overall body size.

While the alpha model does not explicitly incorporate a population genetics model in any statistical analysis, it is possible to further interpret results using simulations and theory (Figure 2). In simulations, the idea is to use a model of choice to generate allele frequencies and effect sizes and then use the inference procedure to estimate what *α* corresponds to those model parameters. For theory, one derives E[*β* ^2^|*x*] under the selection model and compare this to the approximate alpha model expectation of E[*β* ^2^|*x*] ∝ *x* ^*α*^. Using this approach, Schoech et al. (2019) showed that the inferred *α* depends both on the distribution of fitness effects of new alleles affecting the trait (DFE), and on the average scaling of effect sizes and selection E[*β* ^2^|*s*] ∝ *s* ^2*τ*^, where the parameter *τ* determines the scaling through the relationship E[*β*] ∝ *s* ^*τ*^ (Eyre-Walker, 2010). The DFE dependence enters primarily through a frequency-threshold effect: alleles below the threshold have effect sizes roughly uncorrelated with frequency because, while above the dependence scales approximately like E[*β* ^2^|*x*] ∝ *x* ^−2*τ*^. The threshold is the frequency below which most new mutations from the DFE are still mostly affected by drift rather than selection, and is therefore lower for a heavy-tailed DFE with a high average *s*. Zeng et al. (2021) used population genetic simulations to fit the DFE for various traits by conditioning on the values they had estimated for *α*, polygenicity, and SNP heritability. They found greater variation in the DFE among trait categories than variation in *α* estimates. *α* estimates that are relatively insensitive to the DFE are consistent with the predictions of Schoech et al. (2019) if most SNPs included in the analysis are above the frequency threshold where selection is detectable. These simulations therefore also demonstrate that polygenicity and heritability are informative about the DFE.

Simons et al. (2018) developed a model for the relationship between effect sizes and selection coefficients based on isotropic stabilizing selection and Fisher’s geometric model (the specifics of the model is discussed in a subsequent section). The number of trait dimensions in this model corresponds to the effective number of independent axes of genetic variation, a value that can be interpreted as the degree of pleiotropy. With a single dimension the selection coefficient is the same as in the classical model of one dimensional stabilizing selection: $|\beta |=\sqrt{2\;sV_{S}}$ . When the number of traits becomes large the relationship becomes *β* ∼ *N* (0,(*V*_*S*_/*n*_*e*_)*s*), where *n*_*e*_ is the effective number of traits, and expressions for moderate pleiotropy interpolate between these extremes. Rather than fit this model to the heritability explained by different minor allele frequencies, Simons et al. (2018) analyzed the distribution of variance contributions, *v* = 2*β* ^2^ *x*(1 − *x*), among genome-wide significant SNPs. For a given mean among discovered loci, the variance of *v* is higher with greater pleiotropy (*n*_*e*_), with a parametric likelihood derived by the authors. The high-pleiotropy model was found to fit the distribution of GWAS hits for standing height and BMI better than the no- and low-pleiotropy alternatives.

Zeng et al. (2021) also simulated varying degrees of pleiotropy using the Simons et al. (2018) model and found that *α* estimates were insensitive to changes in the degree of pleiotropy (*n*_*e*_). This makes sense, given that the alpha model only attempts to fit the average effect size - frequency relationship and suggests that new approaches will be needed to investigate the nature of pleiotropy and the relative importance of the focal trait.

## 4 Model Building Using Geometry and Pleiotropy

Using the distribution of causative allele frequencies and their effects solely on the focal trait, what could be done to further interpret the results of GWAS studies? One advance would be to explicitly include selection in the next generation of models that build upon LDSC or *α* models (Sella and Barton, 2019). We may start by imagining what class of models could fit the joint distribution of (*x*, *β*). Assume a set of parameters Θ that describes the selection model. An analysis would use the likelihood *p*(*x*, *β*|Θ), which decomposes into *p*(*x*, *β*|Θ) = *p*(*x*|*β*, Θ)*p*(*β*|Θ). Since the distribution of effect sizes is not a major concern for selection, inference would focus on *p*(*x*|*β*, Θ). The distribution of frequencies for a given effect size is determined by integrating over the possible fitness effects of a mutation with effect size *β*: *p*(*x*|*β*, Θ) = *∫p*(*x*|*s*, Θ)*p*(*s*|*β*, Θ)*ds*. The effect of selection, *s*, could be either additive or underdominant (stabilizing selection), but could also represent other models beyond these two such as overdominant or fluctuating selection. *x* can be replaced by the age or historical frequency path of the allele (Stern et al., 2021). *p*(*x*|*s*) can be tackled with standard population genetics, so the trickier problem is to provide *p*(*s*|*β*, Θ) in cases of pleiotropic selection.

In an early attempt to do this explicitly, Keightley and Hill (1990) proposed *p*(*s*|*β*, Θ) as the conditional distribution of a two-dimensional Wishart distribution. In this formulation both the mean and variance of *s*, conditional on *β*, are proportional to |*β*| plus a constant, and a correlation parameter determines how the variance scales with the mean. This contrasts with the model of direct stabilizing selection where *s* is proportional to *β* ^2^. Another approach decomposes this distribution as *p*(*s*|*β*, Θ) ∝ *p*(*β*|*s*, Θ)*p*(*s*|Θ). This has the appealing property of separating the link between fitness and trait effects from the distribution of fitness effects (DFE). Eyre-Walker (2010) proposed a form for *p*(*β*|*s*, Θ) where E[*β*] ∝ *s* ^*τ*^ with multiplicative noise. Both of these models try to capture a space of potential relationships between effect size and selection without being over-parameterized. However, it is not actually clear how one should interpret results from either. A weak correlation parameter from the Keightley and Hill (1990) model would perhaps indicate the importance of pleiotropy, but the linear scaling between |*β*| and *s* would not make sense with direct stabilizing selection. Eyre-Walker’s *τ* doesn’t necessarily mean stronger or weaker selection. Would it mean anything for the relative importance of directional or stabilizing selection? Moreover, these two models make divergent predictions for the contribution of rare versus common alleles to the genetic variance (Eyre-Walker, 2010; Caballero et al., 2015; Sella and Barton, 2019).

Simons et al. (2018) made a strong argument for interpretability when deriving their distribution for *p*(*β*|*s*, Θ). The framework they used was multivariate stabilizing selection in a geometric model (Fisher, 1930). Models within this framework generally posit a multidimensional phenotypic space with a selection function that describes the fitness of each possible phenotypic combination. Typically, the fitness function is Gaussian and centered at some optimum phenotype. A mutation is a vector that moves an individual to a different point in phenotype space, thereby altering fitness. Assuming a population centered at its optimum value, with each phenotypic direction under equal stabilizing selection and mutational pressure, *p*(*β*|*s*, Θ) takes a simple parametric form depending only on the number of dimensions *n*_*e*_ and the strength of selection *V*_*s*_.

Previous work using Fisher’s geometric model had used it to derive the DFE of new mutations (Martin and Lenormand, 2006; Lourenço et al., 2011) or the expected genetic variance and correlation of the focal trait with fitness (Zhang and Hill, 2003) rather than *p*(*β*|*s*). A major assumption of these studies was that the phenotypic effects of new mutations were drawn from a multivariate normal distribution with different dimensions representing different phenotypes. While a seemingly natural starting place, the assumption of normally distributed mutations is far from realistic and mathematically troublesome. There is accumulating evidence that the mutational effect distribution is substantially leptokurtic for many traits (Zhang et al., 2018; O’Connor et al., 2019; O’Connor, 2021). It is has also been shown that, for a single normal distribution of mutations, the DFE concentrates around a point value of *s* as the number of traits becomes large, an obviously unrealistic scenario (Waxman and Peck, 1998; Wingreen et al., 2003; Zhang and Hill, 2003). Thankfully, one may still rescue the utility of geometric models by using a mixture of normals.

For example, the Simons et al. (2018) likelihood can be derived from the geometric model with normal mutation proposed by Martin and Lenormand (2006) by integrating out a variance parameter: $p\left(s|\beta \right)=\int \frac{p\left(\beta |s,\sigma^{2}\right)p\left(s|\sigma^{2}\right)}{p\left(\beta |\sigma^{2}\right)}p\left(\sigma^{2}\right)d\sigma^{2}$ . If mutations are uncorrelated, equally affected by stabilizing selection, and drawn from a mixture of normal distributions, then the distribution of variances, *p*(*σ* ^2^), fully describes the mutational distribution. It is straightforward using Bayes’ theorem to show that *p*(*s*|*β*) ∝ *p*(*β*|*s*)*∫p*(*s*|*σ* ^2^)*p*(*σ* ^2^)*dσ* ^2^ and contains two components. The first component, *p*(*β*|*s*), has the form derived by Simons et al. (2018), a normal distribution with variance proportional to *s* when the number of traits is large. This part is independent of *σ* ^2^. The second component, *∫p*(*s*|*σ* ^2^)*p*(*σ* ^2^)*dσ* ^2^, is the DFE itself. In this example, the DFE is generated by the distribution of mutational effects. The variance of what normal distribution in the mixture a mutation comes from determines how strongly selected it is.

The above approach suggests that a fruitful way to propose future models would be to propose that there exist different mutational modes. Modes might represent different biological pathways and could be parameterized by which traits are involved, the correlation of mutational effects among these, and the distribution of mutational effect sizes. If summarized in Θ_*M*_, we might then integrate over the distribution of modes. If *β* is conditionally independent of *s* given Θ_*M*_, then the form of the DFE will be separable from the link between selection and effect sizes, though it is not always clear that this will be the case. Directional selection as well as antagonistic pleiotropy may be possible to model this way, at least for a population at equilibrium in its fitness landscape. To more directly analyze selection and the pleiotropic relationships among traits, a vector of effect sizes could replace the effect *β* on a single, focal trait.

## 5 Empirical Demonstrations of the Existence and Nature of Pleiotropy

Since models of the evolution and maintenance of complex trait variation strongly depend on assumptions regarding the degree of pleiotropy. Modeling and measurement of pleiotropy is key to the empirical questions of whether the focal trait is under meaningful direct selection and how selection coefficients depend on the phenotypic effects of individual variants.

Current estimates of polygenicity indirectly but strongly suggest highly pleiotropic genetic architecture for most complex traits (Zeng et al., 2018; O’Connor et al., 2019; Zeng et al., 2021). Indeed, it was estimated that 2% of genetic variation is involved in height and a similar proportion (1%) is involved in risk of Type 2 Diabetes (Zeng et al., 2021). It is clear that a model where every quantitative trait locus (QTL) affects just a single trait is, due to the finite nature of the human genome, inconsistent with high polygenicity (defined here as the probability that a variant has a non-zero effect on the focal trait). We do not know exactly what fraction of the genome plays any functional role; comparative and functional genomics produce a range of estimates generally on the order of 0.1 (Rands et al., 2014; Gulko et al., 2015). If 10% of the genome is of any functional importance, and trait-affecting mutations originate from this functional fraction, it clearly cannot harbor independent QTLs for a vast number of complex traits each with a polygenicity of 2% (Jordan et al., 2019).

**FIGURE 3:**
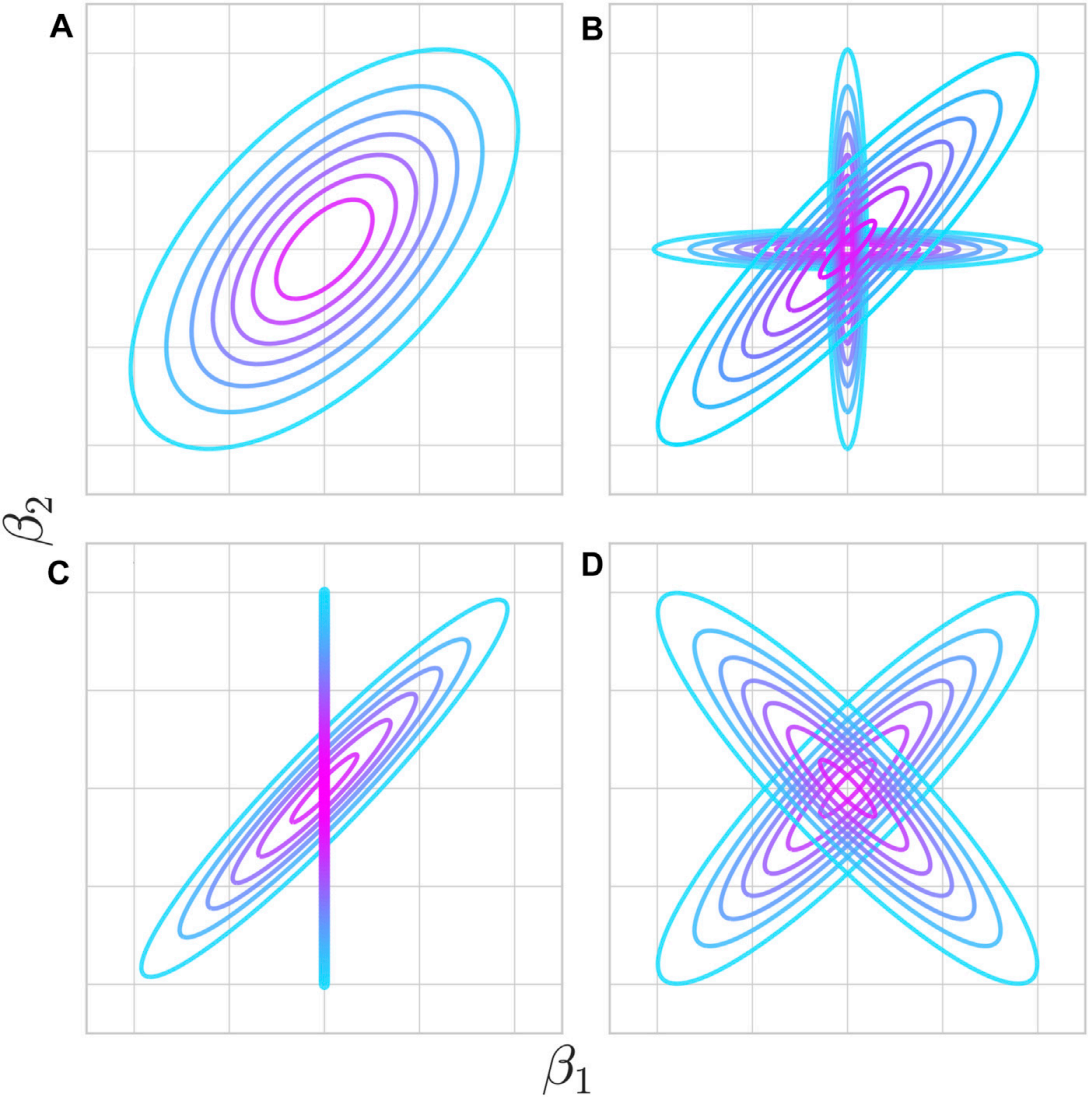
Various potential pleiotropic relationships at individual loci underlie genetic correlations between traits. **(A)** Mutations affecting trait 1 have a tendency to impact trait 2 in a particular direction, although a variety of outcomes are possible through the functional particulars of that change. **(B)** Mutations fall either into a shared or unshared functional pathway between the two traits. Colocalization analysis aims to test which distribution a given QTL comes from. Even though not every mutation is pleiotropic, the two traits are genetically correlated. The proportion of mutations falling into either pathway determines the strength of genetic correlation. **(C)** Trait 1 has a causal impact on trait 2 such that every mutation with a non-zero effect on trait one has a strongly correlated effect on trait 2, but not vice-versa. Mendelian randomization aims to test for the existence and direction of this effect. This also manifests as a genetic correlation at the phenotypic level. **(D)** Individual variants may be pleiotropic, but can result in low or zero genetic correlation if different pathways have opposing effects.

With the abundance of GWAS data, many aspects of pleiotropy can be empirically estimated using corresponding well-developed statistical approaches. The specific relationships between causative QTL effect sizes on different traits that these approaches investigate are illustrated in Figure 3. The relationship between two phenotypes is most commonly expressed as global genetic covariance, which reflects the overall degree of pleiotropy in the form of correlation of QTL effects across all loci (Cov[*β*_1_, *β*_2_], where *β*_1_ and *β*_2_ are allelic effect sizes for phenotypes 1 and 2), scaled by the heterozygosity at each causative locus. A significant genetic covariance below one would indicate that individual QTL effects are correlated but not identical. Global genetic covariance is estimated using statistical approaches related to those used to estimate heritability including random effect models implemented into the GCTA software or LD-score regression (Lee et al., 2012; Bulik-Sullivan et al., 2015). Estimates of genetic covariance come with the same caveats as must apply to heritability estimates (Visscher et al., 2008). Measurements apply to the particular environment in which the different traits are measured and offer no guarantee of a fundamental relationship between traits. Under different conditions, gene-by-environment interactions can change which genetic variants contribute to heritability and different pleiotropic traits may associate with the new regime.

Using these and related statistical techniques, highly significant genetic covariances were estimated among various autoimmune diseases and among various psychiatric diseases and related phenotypes (Cotsapas et al., 2011; Lee et al., 2013; Watanabe et al., 2019; Lincoln et al., 2021). The analysis of genetic covariances between two traits has some limitations. The genetic covariance alone is not informative about biological mechanisms and per locus patterns. For example, the same genetic covariance may indicate either pleiotropy limited to just a few loci with very similar effects on both traits on the background of other non-pleiotropic loci or the broad pleiotropy of all loci but with non-identical effects (Figures 3A vs 3B). For autoimmune traits, it is possible that some loci impact immune function while others determine tissue or organ specificity.

The question of contribution of individual loci into global genetic correlation must be, therefore, addressed at the local level by studying individual loci. When studying individual loci, one of the challenges is that linkage disequilibrium confounds the analysis. Genetic covariance may imply real pleiotropy, meaning that the same genetic variants causally affect both traits. Alternatively, some variants may exclusively impact the first trait and other variants exclusively impact the second trait, but local genetic correlation can still be induced by linkage disequilibrium between the two sets. Consequently, the field has developed two different classes of methods to address this issue. Methods that estimate local genetic covariance (Shi et al., 2017) do not distinguish between functional pleiotropy versus non-independence induced by linkage disequilibrium. A different class of methods called “colocalization” (Giambartolomei et al., 2014; Hormozdiari et al., 2016; Chun et al., 2017) relies on linkage disequilibrium patterns to specifically test the hypothesis that the same causative variant (or variants) in the locus impacts both traits (Figure 3B). Multiple examples of local genetic correlations and individually colocalized loci have been described (van Rheenen et al., 2019; Aguet et al., 2020; Vuckovic et al., 2020). However, some QTLs involved in genetically correlated traits do not show obvious signals of colocalization, suggesting that genetic correlation does not necessarily imply pleiotropic effects of all variants (Lincoln et al., 2021).

A separate aspect of pleiotropy that statistical genetics addresses is the causal relationship between phenotypes (van Rheenen et al., 2019). There is an important distinction between “horizontal” pleiotropy with genetic variants exerting independent effects on both traits and a causal path or “vertical” pleiotropy, where one trait directly contributes to the other (Jordan et al., 2019). Examples of the latter include LDL cholesterol being a causative risk factor of heart disease (Zhu et al., 2018), the genetic component of smoking being a causative risk of lung cancer (McKay et al., 2017), and all molecular effects (considered as “molecular” phenotypes) leading to changes in a phenotype of the organism. If one trait is a cause of the other trait, every variant inducing an effect on the first trait also affects the second trait (Figure 3C). Moreover, these effect sizes are proportional and correspond to the causal effect of the first trait on the second trait. Because the first trait is usually just one of many causes, most variants affecting the second trait would not be expected to have any effect on the first trait. These considerations are a foundation of Mendelian Randomization methods that attempt to infer causal relationships even if genetic associations for the two phenotypes are measured separately in independent datasets (Pingault et al., 2018). This approach relies on a large number of QTLs and does not translate to individual loci.

Many recent studies of pleiotropy, colocalization and causality have focused on molecular phenotypes such as gene expression, chromatin accessibility or DNA methylation (Umans et al., 2020; Vuckovic et al., 2020; Ye et al., 2020; Morabito et al., 2021). Numerous QTLs for various molecular phenotypes have been identified for these classes of traits (most prominently expression QTLs or eQTLs). The main motivation of these studies is to identify the primary molecular events underlying genetic associations with human traits and diseases. However, it is not guaranteed that genetic covariance or colocalization of a molecular trait with a focal trait is indicative of an underlying causal impact of variation in the molecular trait on the focal trait. One example is that changes in BMI actually induce changes in DNA methylation rather than DNA methylation acting as a molecular mechanism mediating genetic effects on BMI (Wahl et al., 2017). The direction of causality was demonstrated by showing that SNPs which predict methylation levels at individual loci did not predict BMI levels, while a genetic risk score for BMI levels did predict methylation levels.

Even in the absence of genetic covariance, molecular effects may induce pleiotropic relationships between two traits. Imagine a scenario where the two traits are both mediated by a large number of molecular phenotypes (activities of many individual genes or other latent factors), but these molecular phenotypes do not exhibit correlated effects on the two traits (Figure 3D). In this case, genetic covariance might not exist or be very weak on aggregate but covariance between absolute (or squared) genetic effects $\text{Cov}\left[\beta_{1}^{2},\beta_{2}^{2}\right]$ may be substantial. The popular “omnigenic” model offers one version of such a scenario (Boyle et al., 2017). Genetic covariance may also be close to zero if the pleiotropic effect is limited to one or a small number of loci (in other words, with a substantial local genetic covariance and even colocalization in individual loci) (Liu et al., 2019).

These methodological developments and empirical results related to pleiotropy are important in light of the main subject of this review. They motivate consideration of evolutionary models that take into account groups of correlated traits. For causally related traits, selection effects would probably differ depending on whether selection primarily acts on the upstream or downstream trait along the causal chain. An interesting perspective is also brought by the consideration of molecular phenotype. If each molecular phenotype is pleiotropically involved with many downstream organismal phenotypes, and the focal trait is merely one of these, selection coefficients can depend on effect sizes even if the focal trait is neutral. Variants with larger effect sizes on molecular function would be under stronger selection because this molecular function impacts multiple other selected downstream traits in addition to the neutral focal trait.

Few studies have analyzed the effects of pleiotropy on selection by actually incorporating the measured effects of variants on multiple traits. Some mutation accumulation studies have tried to demonstrate whether pleiotropic mutations are under stronger selection. McGuigan et al. (2014) provide some evidence that mutations underlying combinations of correlated gene expression traits in Drosophila serrata are under stronger selection than the average mutation affecting a given trait. In humans, Sanjak et al. (2018) regressed lifetime reproductive success on genetic scores for multiple traits simultaneously in United Kingdom Biobank participants. Compared to the univariate, this full analysis lacked power, but quadratic and linear selection terms did change in both magnitude and sign for some traits, indicating the importance of accounting for pleiotropy. Stern et al. (2021) took a similar approach, but used the shape of genealogies at GWAS loci to look at historical rather than contemporary patterns of directional selection. Again, the authors found that many estimates of selection changed substantially, and were largely attenuated, when accounting for the correlated response in other traits. At the time of writing, no attempt has been made to account for pleiotropy in the alpha model approaches discussed above that have demonstrated negative selection on many human traits.

## 6 Constancy of Effect Sizes Across Time and Space

Everything discussed so far has assumed that genetic variants have well-defined additive effects on traits of interest, and that these effects are measurable in contemporary human populations. Although convenient, and the correct starting place for most analyses, recent research has demonstrated that causative variants for many traits and diseases have population-specific effect sizes. Such studies are possible when GWAS for the same traits have been performed in different populations (De Candia et al., 2013; Mancuso et al., 2016). One approach has been to estimate the cross-population genetic correlation, the correlation in causal effect sizes between the different samples, and these estimates are often less than one (Brown et al., 2016; Galinsky et al., 2019). This is most likely due to gene-by-environment (GxE) and gene-by-gene (GxG) interactions, with some effect driven by different measurement practices and diagnosis criteria.

Shi et al. (2021) estimated the impact of different functional annotations on the degree of cross-population effect size correlation of variants within those genomic regions. They found that the squared genetic correlation was depleted most strongly in regions under strong background selection as well as in and around functional elements such as exons, promoters, and enhancers. These regions are also enriched for heritability and, as previous research reviewed here has shown, variants residing there are likely under stronger selection. If a variant has different effect sizes in different contemporary populations, we should be more uncertain about its effect size in the ancestral population where the majority of its existence may have taken place. Cross-population genetic correlation could therefore be used as a measure of the temporal stability of allelic effects. Alternatively, the aggregate pleiotropic effects of an allele may stay roughly constant even as the effects on individual traits vary due to GxE or other factors.

## 7 Conclusion

Direct data on genotype-phenotype associations for numerous human traits have provided an opportunity to investigate which, if any, of the current theoretical models for the maintenance of complex trait variation fit observed genetic architectures. Depending on the degree and nature of pleiotropy, as well as the importance of the focal trait for selection, these models predict the relationship between *β* and *s* (Johnson and Barton, 2005). Selection analyses of human GWAS data have consistently demonstrated a negative relationship between effect size magnitudes and allele frequencies, implying that larger effect sizes are associated with stronger selection on average (Zeng et al., 2018; Schoech et al., 2019; Speed et al., 2020; Zeng et al., 2021). Models where the focal trait is neutral, or largely biologically unrelated to any aspect of fitness, are therefore ruled out. Within the class of alpha models, the scaling between *β* ^2^ and frequency varies across traits, likely reflecting differences in the DFE and the scaling between *β* and *s*. These estimates are difficult to interpret in terms of classical stabilizing selection models, and work is needed to reconcile tractable statistical models of how effect sizes change with frequency with realistic models of selection at the phenotypic level. Studies have also largely been limited to analyzing the average relationship of effect size to frequency. This limits the ability to capture the importance of pleiotropy which should create variance around that average. By directly modeling the variation in genome-wide significant variance contributions, Simons et al. (2018) were able to infer a high degree of pleiotropy for height and BMI.

All the approaches reviewed here infer the nature of selection on GWAS loci by analyzing the distribution of allele frequencies and effect sizes (*x*, *β*), with the overall trait heritability sometimes included. Future work along these lines may utilize fine-mapping (Weissbrod et al., 2020) or other techniques to better capture this distribution (O’Connor, 2021). An interesting approach was developed by O’Connor et al. (2019) who estimated the kurtosis of heritability contributions using the LDSC framework to measure trait polygenicity at different allele frequencies and functional genomic annotations. The kurtosis depends on the fourth moment of the distribution of effect sizes, and therefore contains additional information beyond that contained in the alpha model. A low kurtosis, and therefore high polygenicity, of common variants indicated a “flattening” due to selection strongly preventing any large-effect variants from reaching high frequencies. This indicates a high importance of the focal trait for selection, but more thought is needed to tell what degree of pleiotropy is consistent with these results.

The greatest advances in our ability to make sense of the maintenance of complex trait variation will likely come from analyses that utilize variant-level pleiotropy and account for effect sizes that vary across time and space. Methods to investigate pleiotropy in statistical genetics are already well-developed (van Rheenen et al., 2019) but have yet to intersect with analyses of stabilizing or negative selection. Effect size differences between populations are also well-documented (Brown et al., 2016; Shi et al., 2021), but have received less attention, likely in part due to the lack of large GWAS from diverse populations and the difficulty of standardizing measurement for some phenotypes. The portability of polygenic scores is also potentially more strongly impacted by differences in allele frequencies and linkage disequilibrium than effect size variation (Wang et al., 2020), and allele frequencies will differentiate more rapidly under stabilizing or negative selection (Yair and Coop, 2021). However, understanding effect size variation in space and time may ultimately end up being more important for modeling the maintenance of variation in complex traits as well as detecting selection on them (Mathieson, 2021).

## Glossary

Causative Allele: The allele which causally affects the focal trait. Due to linkage disequilibrium, many alleles at a GWAS-identified locus are highly correlated. The causative allele refers only to the one which causally affects the trait.
Distribution of Mutational Effects: The distribution from which the phenotypic effects of new mutations are drawn. This can include the focal trait as well as related pleiotropic ones.
Effect Size Magnitude: The absolute value of the effect size of an allele. It is often useful to ignore the direction of effect that an allele has on the trait.
Focal Trait: All studies in quantitative genetics must choose some measurable aspects of biology to focus on. This can be something of obvious importance like diabetic status, or could be simply something easily queried in a biobank. Analyzed one at a time, we call the current trait the focal trait.
Genetic Architecture: The joint distribution of allele frequencies and effect sizes in a population or sample. This determines how much different frequency and effect size ranges contribute to heritability, and answers questions surrounding the importance of rare versus common variants; genetic covariance, The covariance between the effects different genotypes have on two traits. This measures the propensity for an individual with a high genetic value for one trait to also have a high (or low) genetic value for the second. It averages over all alleles and their effects on both traits, scaled by their contributions to the genetic variance.
Genetic Risk Score: A phenotypic prediction calculated for an individual using a weighted sum of the estimated effect sizes of variants found in that individuals genome.
Molecular Phenotype: Phenotypes such as gene expression, methylation levels, or metabolite concentration that are measured at the molecular level. These are hoped to represent “low-level” traits that mediate the effects of genetic variants on other phenotypes.
Overdominant: Selection where the heterozygous genotype has higher average fitness than either of the two homozygotes.
Pairwise Coalescent Time: The amount of time it takes two sampled loci to find a common ancestor going backwards in time. The longer this time, the more likely it is that mutations occur to differentiate the two loci.
QTL: Quantitative trait locus. A region in the genome that has been statistically associated with a quantitative trait.
